# A commonly occurring genetic variant within the *NPLOC4–TSPAN10–PDE6G* gene cluster is associated with the risk of strabismus

**DOI:** 10.1007/s00439-019-02022-8

**Published:** 2019-05-09

**Authors:** Denis Plotnikov, Rupal L. Shah, Jamille N. Rodrigues, Phillippa M. Cumberland, Jugnoo S. Rahi, Pirro G. Hysi, Denize Atan, Cathy Williams, Jeremy A. Guggenheim, UK Biobank Eye and Vision Consortium

**Affiliations:** 1 School of Optometry and Vision Sciences, Cardiff University, Cardiff, CF24 4HQ UK; 2 Population Health Sciences, Bristol Medical School, University of Bristol, 1-5 Whiteladies Road, Bristol, BS8 1NU UK; 3 Life Course Epidemiology and Biostatistics Section, Institute of Child Health, University College London, London, WC1N 1EH UK; 4 Ulverscroft Vision Research Group, University College London Institute of Child Health, London, WC1N 1EH UK; 5 University College London Great Ormond Street Institute of Child Health, London, WC1N 3JH UK; 6 National Institute for Health Research Biomedical Research Centre at Moorfields Eye Hospital NHS Foundation Trust and University College London Institute of Ophthalmology, London, WC1E 6BT UK; 7 Department of Twin Research and Genetic Epidemiology, King’s College London, St Thomas’ Hospital, London, SE1 7EH UK

## Abstract

Strabismus refers to an abnormal alignment of the eyes leading to the loss of central binocular vision. Concomitant strabismus occurs when the angle of deviation is constant in all positions of gaze and often manifests in early childhood when it is considered to be a neurodevelopmental disorder of the visual system. As such, it is inherited as a complex genetic trait, affecting 2–4% of the population. A genome-wide association study (GWAS) for self-reported strabismus (1345 cases and 65,349 controls from UK Biobank) revealed a single genome-wide significant locus on chromosome 17q25. Approximately 20 variants across the *NPLOC4–TSPAN10–PDE6G* gene cluster and in almost perfect linkage disequilibrium (LD) were most strongly associated (lead variant: rs75078292, OR = 1.26, *p *= 2.24E−08). A recessive model provided a better fit to the data than an additive model. Association with strabismus was independent of refractive error, and the degree of association with strabismus was minimally attenuated after adjustment for amblyopia. The association with strabismus was replicated in an independent cohort of clinician-diagnosed children aged 7 years old (116 cases and 5084 controls; OR = 1.85, *p *= 0.009). The associated variants included 2 strong candidate causal variants predicted to have functional effects: rs6420484, which substitutes tyrosine for a conserved cysteine (C177Y) in the *TSPAN10* gene, and a 4-bp deletion variant, rs397693108, predicted to cause a frameshift in *TSPAN10*. The population-attributable risk for the locus was approximately 8.4%, indicating an important role in conferring susceptibility to strabismus.

**Electronic supplementary material:**

The online version of this article (10.1007/s00439-019-02022-8) contains supplementary material, which is available to authorized users.

## Introduction

Strabismus refers to an abnormal alignment of the eyes leading to loss of binocular vision. Concomitant strabismus occurs when the angle of deviation is constant in all positions of gaze and often manifests in early childhood when it is considered to be a neurodevelopmental disorder of the visual system. Concomitant strabismus is most often convergent (‘esotropia’) or divergent (‘exotropia’), although vertical misalignment may also occur as the primary deviation or in conjunction with eso- or exotropia. It is often associated with amblyopia (defined as poor visual acuity in one or both eyes not immediately correctable by glasses and without accompanying ocular pathology) in the deviated eye—either because the squint itself leads to secondary amblyopia in the deviated eye or because reduced vision in one eye compared with the other (e.g. anisometropia, unilateral cataract) secondarily leads to the squint. Therefore, concomitant strabismus is frequently associated with childhood-onset visual impairment, usually in one eye (Robaei et al. 2006).

A variety of prenatal and early life environmental factors, such as prematurity, maternal smoking, and ill-health during pregnancy, increase the risk of strabismus, as does a high hyperopic refractive error in early childhood (Cotter et al. 2011; Pathai et al. 2010; Atkinson et al. 1996). Numerous studies have been conducted to understand the genetics of strabismus (Graeber et al. 2013; Kruger et al. 2013; Maconachie et al. 2013). Despite a complex inheritance pattern (Sanfilippo et al. 2012; Ye et al. 2014; Georges et al. 2013), linkage analysis in pedigrees with multiple affected participants has implicated a locus at 7p22.1 (strabismus, susceptibility to, 1; STBMS1) consistent with the idea that rare, monogenic subtypes may exist (Parikh et al. 2003; Rice et al. 2009). Strabismus is also a feature of several rare syndromes—often in conjunction with intellectual disability—including examples such as Mietens–Weber syndrome and Lamb–Shaffer syndrome.

Recently, Shaaban et al. (2018) tested the hypothesis that commonly occurring polymorphisms in the general population increase the risk of non-syndromic strabismus, by carrying out a genome-wide association study (GWAS) for strabismus. They identified a locus within the first intron of *WRB* (tryptophan-rich basic protein) on chromosome 21 (lead variant rs2244352; OR = 1.33, *p *= 9.58E−11). Here, we sought to test the same hypothesis in a larger sample, in order to increase statistical power. In view of the known association between strabismus and hyperopia, the refractive error of participants was included as a covariate in our GWAS analysis to avoid detecting genetic variants that increase the risk of strabismus secondary to hyperopia. The advent of the UK Biobank project facilitated this work, by providing a larger sample of cases with strabismus than has been studied before.

## Materials and methods

### Phenotypes in UK Biobank participants

Approximately 500,000 individuals aged 37–73 years were recruited into the UK Biobank cohort study between February 2006 and July 2010 (Sudlow et al. 2015). Ethical approval was obtained from the National Health Service National Research Ethics Service (Ref 11/NW/0382) and all participants provided written informed consent. At a baseline visit to 1 of 22 assessment centres across the UK, participants completed a touch-screen questionnaire and underwent a series of physical assessments. From 2009, the physical assessments included an ophthalmic examination (Cumberland et al. 2016). Approximately 23% of participants underwent the ophthalmic assessment at the baseline visit, while an additional small proportion completed this assessment at the first of up to two follow-up visits. Based on their reason for wearing spectacles or contact lenses at the baseline or first follow-up visit [field #6147], participants were classified as having self-reported strabismus. Specifically, participants were asked ‘Why were you prescribed glasses/contact lenses? (You can select more than one answer)’. The response options were: (1) ‘For short-sightedness, i.e. only or mainly for distance viewing such as driving, cinema, etc., (called “myopia”)’; (2) ‘For long-sightedness, i.e. for distance and near, but particularly for near tasks like reading (called “hypermetropia”)’; (3) ‘For just reading/near work as you are getting older (called “presbyopia”)’; (4) ‘For “astigmatism”; (5) ‘For a “squint” or “turn” in an eye since childhood (called “strabismus”)’; (6) ‘For a “lazy” eye or an eye with poor vision since childhood (called “amblyopia”)’; (8) ‘Other eye condition’, or (7) ‘Do not know’. Note that the age-of-onset, and the type of strabismus, for example esotropia or exotropia, was not ascertained during the UK Biobank assessment, hence all subtypes would have been captured within the ‘self-reported strabismus’ phenotype. Similarly, information on strabismus surgery in childhood was not available (since data on hospital in-patient operations were collected only from April 1997 onwards). In addition, participants who had strabismus, but who were not prescribed glasses/contact lenses because of the condition, would have been categorised as ‘controls’ rather than ‘cases’ for this self-reported strabismus phenotype.

Non-cycloplegic refractive error was measured after removal of habitual spectacles or contact lenses using a Tomey RC5000 autorefractor [fields #5084, #5085, #5086 and #5087]. All repeat readings were averaged after removal of those flagged as unreliable [fields #5090 and #5091]. Spherical equivalent refractive error was calculated as sphere power plus half the cylinder power. The refractive error of an individual was taken as the average spherical equivalent of the two eyes (Tedja et al. 2018). Myopia was defined as a spherical equivalent refractive error ≤ − 0.50 diopters (D). Refractive astigmatism was taken as the average cylinder power between the two eyes. A binary variable was used to classify individuals with astigmatism ≥ 1.00 D (Shah et al. 2018). Anisometropia was calculated as the difference in spherical equivalent between the two eyes. A binary variable was used to classify individuals with anisometropia ≥ 1.00 D (Qin et al. 2005).

### Genetic data in UK Biobank participants

UK Biobank carried out DNA extraction from blood samples, and obtained high-density genotypes using either the UK BiLEVE array (*n* = 49,950) or the UK Biobank Axiom array (*n* = 438,427). Prior to release of the genetic data (“July 2017 release”), Bycroft et al. (2018) performed extensive quality control of the genotype data and imputed additional genotypes using the HRC reference panel (McCarthy et al. 2016). Imputed genotype data were available for 488,377 individuals. Participants who had withdrawn consent were removed, as were those not in the White British European ancestry subset defined by Bycroft et al. (2018), those whose genetic sex did not match their self-reported gender, and those with heterozygosity more than 4 standard deviations from the mean. A set of well-imputed variants (with IMPUTE2 INFO metric > 0.9, MAF > 0.005, missing rate ≤ 0.01, and an ‘rs’ variant ID prefix) were selected and LD-pruned using the --indep-pairwise 50 5 0.1 command in PLINK 2.0 (Chang et al. 2015). These were used to create a genetic relationship matrix (GRM) with PLINK 2.0 in order to identify a set of unrelated individuals (command --rel-cutoff 0.025). Note that phenotype information was not taken into account when selecting a set of unrelated participants. This left 338,253 unrelated participants of White British ancestry.

**Fig. 1:**
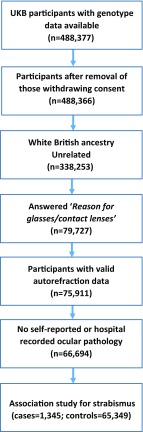
Flow diagram illustrating the selection of UK Biobank participants for the GWAS sample

As shown in Fig. 1, further exclusions were made for participants who: did not have a response for the questionnaire item [field #6147] ‘Why were you prescribed glasses/contact lenses?’; did not have valid autorefraction information; self-reported a history of laser refractive surgery [field #5325], cataract surgery [field #5324; #20004; ICD 1435], corneal graft surgery [field #5328], any other eye surgery in the last 4 weeks [field #5181], any eye trauma resulting in sight loss [#5419, #6148], serious eye problems [field #6148], or self-report of having cataracts [fields #5441, #6148; #20004; ICD 1278] or retinal detachment [field #20004; ICD 1281]. Participants were also excluded if their hospital records [field #41200] indicated they had undergone cataract surgery [OPCS-4: C711, C712, C718, C719, C723, C751], retinal detachment surgery [OPCS-4: C548, C549, C543, C545], or corneal surgery [OPCS-4: C442, C444, C445, C448, C461, C462, C463, C465, C493]. The final UK Biobank ‘discovery sample’ comprised 66,694 participants.

### Genome-wide association study for strabismus in UK Biobank participants

A GWAS for self-reported strabismus was carried out for the 1345 cases and 65,349 controls in the discovery sample. Firth logistic regression was performed (--glm-firth command in PLINK 2.0) for 7,469,170 imputed variants in the HRC reference panel with MAF ≥ 1%, INFO > 0.8, missing genotype rate < 1.5%, and HWE *p* value > 1.0e−06. Only individuals with a missing genotyping rate < 2.5% were included. Age, gender, refractive error averaged between the two eyes, genotyping array (UK BiLEVE or UK Biobank Axiom, coded as a binary variable), and the first 10 ancestry PCs were included as covariates.

### Post-GWAS analyses

Fine-mapping of the *NPLOC4*–*TSPAN10*–PDE6G region was carried out using FINEMAP v1.3 (Benner et al. 2016). All SNPs on the HRC reference panel within ± 250 kb of the lead variant rs75078292 or that were in LD (*r*^2^ > 0.1) with the lead variant were included in the analysis. Up to 5 causal variants in the region were considered, with a prior probability within the range 0.583–0.005 of having 1–5 causal variants. The Genotype-Tissue Expression consortium (GTEx) Portal (The GTEx Consortium 2015) was accessed on 09/18/2018 to identify expression quantitative trait loci (eQTL) for genes situated at the locus regulated by the lead SNP identified in the GWAS (so-called ‘eGenes’).

### Statistical analyses and population-attributable risk

Statistical tests were performed using R (R Development Core Team 2008). Standard logistic regression models were fitted using the *glm* function, while Firth regression models were fitted using the *brglm* function from the *brlrmr* package. A likelihood ratio test was used to compare the fit of additive vs. dominant or additive vs. recessive models (Bagos 2013). This was accomplished by deriving dummy variables and fitting nested models, as described in Online Resource 1. In view of the low prevalence (2–4%) of strabismus, we made the assumption that the relative risk was approximately equal to the odds ratio (OR), and therefore the population-attributable risk (PAR) due to the lead GWAS variant was estimated with the formula (Qi et al. 2011): PAR = *p* (OR−1)/[*p* (OR−1)] + 1, where *p* is the proportion of controls with the risk genotype. The IMPUTE2 INFO score for the lead GWAS variants rs75078292 was 0.999, suggesting that there was no loss in accuracy by using ‘hard’ genotype calls in the above models rather than dosage accounting for genotype uncertainty.

### ALSPAC replication sample

The Avon Longitudinal Study of Parents and Children (ALSPAC) (Boyd et al. 2013; Fraser et al. 2012) is a birth cohort study that recruited 14,541 pregnant women resident in Avon, UK, with expected dates of delivery 01/04/1991–31/12/1992. Of these initial pregnancies, 13,988 children were alive at 1 year of age. When the oldest children were approximately 7 years of age, an attempt was made to bolster the initial sample with eligible cases who had failed to join the study originally. This resulted in an additional 811 children joining the study. Ethical approval for the study was obtained from the ALSPAC Ethics and Law Committee and the Local Research Ethics Committees. Please note that the study website (http://www.bristol.ac.uk/alspac/researchers/our-data/) contains details of all the data that are available through a fully searchable data dictionary and variable search tool.

DNA extraction, genotyping and imputation of ALSPAC participants has been described previously (Taylor et al. 2016). Imputed genotype data were available for a total of 8237 children. Eye movements were assessed by an orthoptist when ALSPAC participants were aged 7 years old (Williams et al. 2008). Ocular misalignment was quantified by simultaneous prism cover test and alternate prism cover test, at near (33 cm) and distance (6 m) with and without glasses, if worn. Strabismus was classified as ‘manifest’ if present in normal viewing with both eyes open. Horizontal strabismus was categorised as esotropia (convergent) or exotropia (divergent) and included manifest and large latent deviations (> 10 pd for convergent and > 15 pd for divergent) so as to be more likely to capture intermittent or decompensating cases. Logistic regression was used to examine the association between each ocular phenotype and rs6420484 genotype (coded either as additive or recessive) in the 5200 children with genotype and ocular phenotype data available. The phenotypes examined were: parentally reported history of strabismus (*n* = 145 cases), manifest strabismus (*n* = 116 cases), esotropia (*n* = 143 cases) and exotropia (*n* = 28 cases).

### Retinal immunohistochemistry

Immunostaining of adult mouse retinal sections (> 8 weeks old) was performed as previously described (Jung et al. 2015). Antibodies were used at the following dilutions: peanut agglutinin conjugated to Alexa 488 (1:500), mouse anti-rhodopsin (1:5000), rabbit anti-TSPAN10 (1:1000), mouse anti-GOalpha (1:500), rabbit NPLOC4 (1:10), and mouse anti-PKC (1:1000). Images were acquired using a Leica laser scanning confocal microscope.

## Results

### Validation of self-reported strabismus

**Fig. 2:**
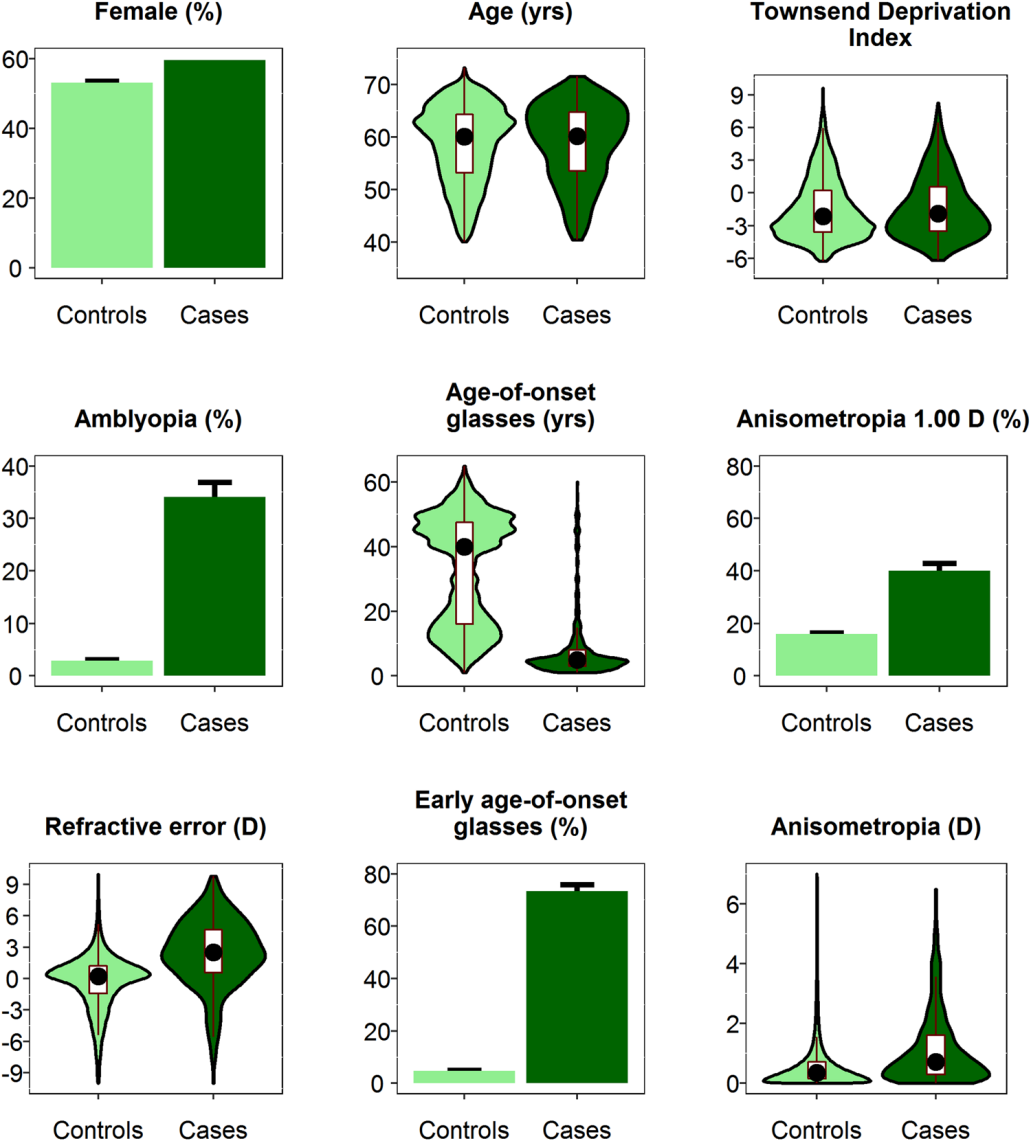
Clinical and demographic characteristics of participants self-reporting strabismus compared to controls (*n* = 1345 cases and *n* = 65,349 controls). Early age-of-onset of glasses was defined as ≤ 7 years. Bar chart error bars denote 95% CI. For violin plots, the white rectangle corresponds to the interquartile range and the solid black circle to the median

There were 72,911 unrelated UK Biobank participants who reported their country of birth as England, Wales or Scotland, whose genetic ancestry principal components clustered with other White British Europeans, and who had responded to the questionnaire item ‘Why were you prescribed glasses/contact lenses? (You can select more than one answer)’ as well as undergoing an autorefraction assessment. After further removal of participants with a history of ocular pathology, this left 66,694 individuals (Fig. 1). A total of 1345 (2.0%) of participants reported ‘squint or a turn in an eye since childhood’ as a reason for wearing glasses or contact lenses. Participants with self-reported strabismus had a 11.3-fold greater prevalence of self-reported amblyopia, a 2.5-fold greater prevalence of 1.00 D or more anisometropia, a much more hypermetropic refractive error (median + 2.46 vs. + 0.21 D), and a much earlier age of starting to wear glasses or contact lenses (median 5 vs. 40 years old) (Table 1; Fig. 2). For age and gender, the cases and controls were well-matched (Table 1), and the difference in Townsend Deprivation Index, a measure of socioeconomic position, in those who did vs. did not report having strabismus was modest (− 1.84 vs − 2.12; *p* = 4.40E−03). All of these comparisons between cases and controls supported the validity of the self-reported strabismus phenotype in the majority of participants.

**Table 1:** Demographic and ocular characteristics of the UK Biobank strabismus GWAS sample

Variable		All (*n* = 66,694)	Cases (self-reported strabismus; *n* = 1345)	Controls (*n* = 65,349)	*p* value
Female	*N* (%)	35,758 (53.6%)	803 (59.7%)	34,955 (53.5%)	2.20E−01
Self-reported unilateral amblyopia	*N* (%)	2465 (3.7%)	471 (35.0%)	1994 (3.1%)	< 1.0E−99
Both eyes VA ≤ 0.0 logMAR	*N* (%)	30,098 (45.1%)	266 (19.8%)	29,832 (45.6%)	< 1.0E−99
VA difference ≥ 0.2 logMAR	*N* (%)	13,135 (19.7%)	624 (46.4%)	12,511 (19.1%)	< 1.0E−99
Better VA ≤ 0.0; VA difference ≥ 0.2	*N* (%)	9123 (13.7%)	454 (33.8%)	8669 (13.3%)	< 1.0E−99
Anisometropia ≥ 1.0 D	*N* (%)	11,156 (16.7%)	553 (41.1%)	10,603 (16.2%)	< 1.0E−99
Anisometropia ≥ 2.0 D	*N* (%)	3948 (5.9%)	261 (19.4%)	3687 (5.6%)	< 1.0E−99
Age	Median (IQR)	60.17 (53.33 to 64.42)	60.17 (53.67 to 64.83)	60.17 (53.33 to 64.42)	2.30E−01
Refractive error (D) average of 2 eyes	Median (IQR)	0.23 (− 1.41 to 1.26)	2.46 (0.52 to 4.65)	0.21 (− 1.44 to 1.22)	< 1.0E−99
Anisometropia (D)	Median (IQR)	0.36 (0.16 to 0.73)	0.75 (0.30 to 1.67)	0.35 (0.15 to 0.72)	8.00E−94
Age started wearing glasses (years)	Median (IQR)	39.00 (15.00 to 47.00)	5.00 (3.00 to 8.00)	40.00 (16.00 to 47.50)	< 1.0E−99
Townsend Deprivation Index	Median (IQR)	− 2.12 (− 3.59 to 0.25)	− 1.84 (− 3.46 to 0.63)	− 2.12 (− 3.59 to 0.25)	4.40E−03

### Genome-wide association study for self-reported strabismus identifies a locus on 17q25.3

Standard logistic regression is susceptible to bias in highly unbalanced case–control studies, which can result in either conservative or anti-conservative test behaviour (Ma et al. 2013). We therefore carried out our GWAS analysis using Firth bias-corrected logistic regression. A total of 7,469,170 genetic variants were tested for association with self-reported strabismus in the sample of 1345 cases and 65,349 controls (Fig. 1). As well as standard GWAS covariates such as age, sex and PCs, we included refractive error averaged between the two eyes as an additional covariate in order to mitigate against identifying genetic variants primarily associated with this comorbid trait.

**Fig. 3:**
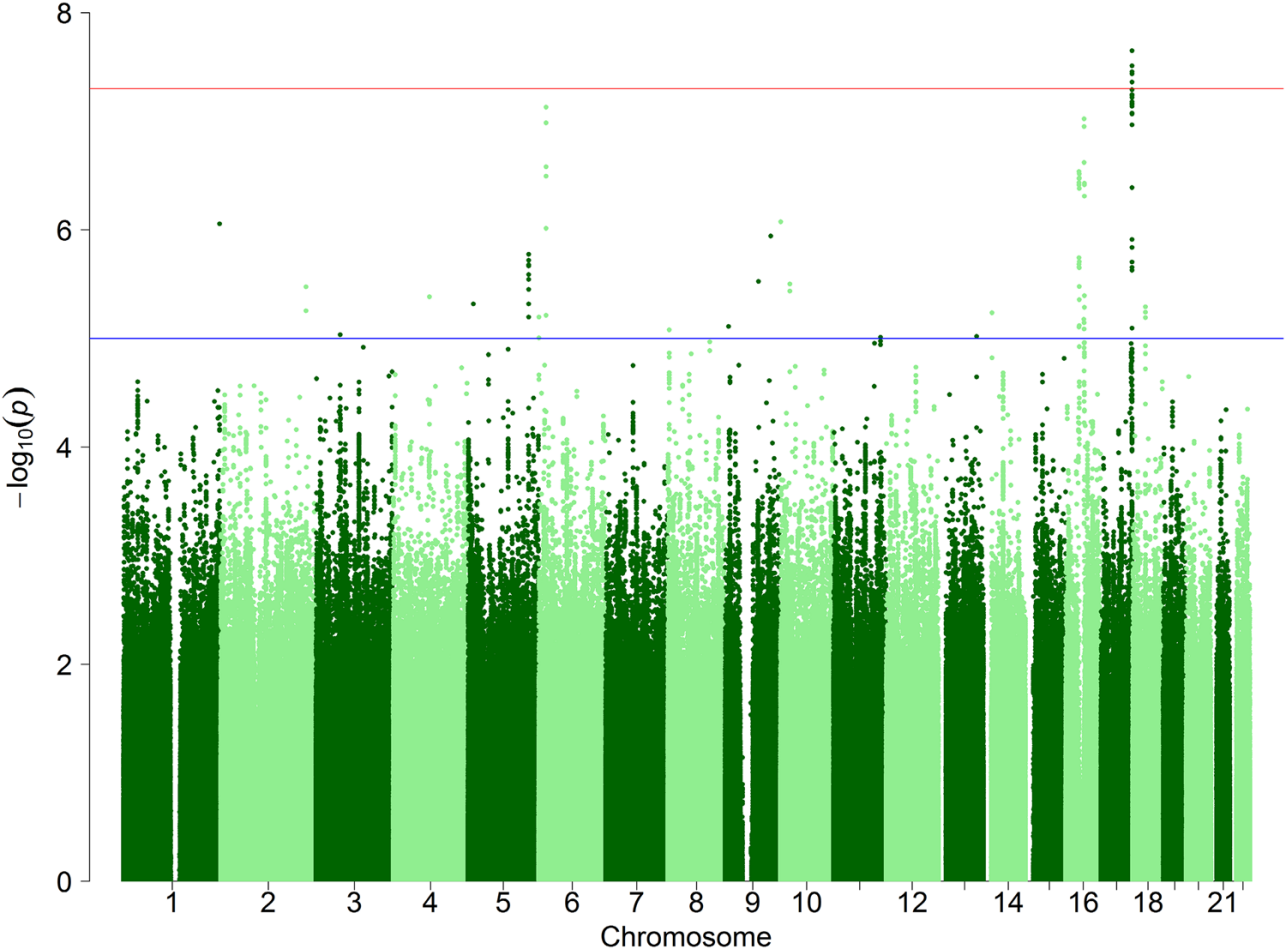
Manhattan plot for self-reported strabismus GWAS. The *y* axis indicates minus log_10_*p* values and the *x* axis genomic position. The red and blue horizontal lines correspond to *p* values of 5.0E−08 and 1.0E−05, respectively

Variants located on chromosome 17q25.3 were found to be significantly associated with strabismus (Fig. 3). The most strongly associated variant was rs75078292, OR = 1.26, 95% CI 1.16–1.36, *p *= 2.24E−08. Markers in other regions did not attain genome-wide significance; the lead variant in all regions that include a marker with *p* < 1.0E−05 are listed in Online Resource 2. The strabismus GWAS analysis showed no evidence of population stratification (*λ*_GC_ = 1.004; Online Resource 3).

The lead variant, rs2244352, identified in the non-accommodative esotropia case–control GWAS reported by Shaaban et al. (2018) was not associated with self-reported strabismus in the current GWAS (OR = 1.01, 95% CI 0.93–1.10, *p *= 0.83).

### Fine-mapping of the 17q25.3 locus

**Fig. 4:**
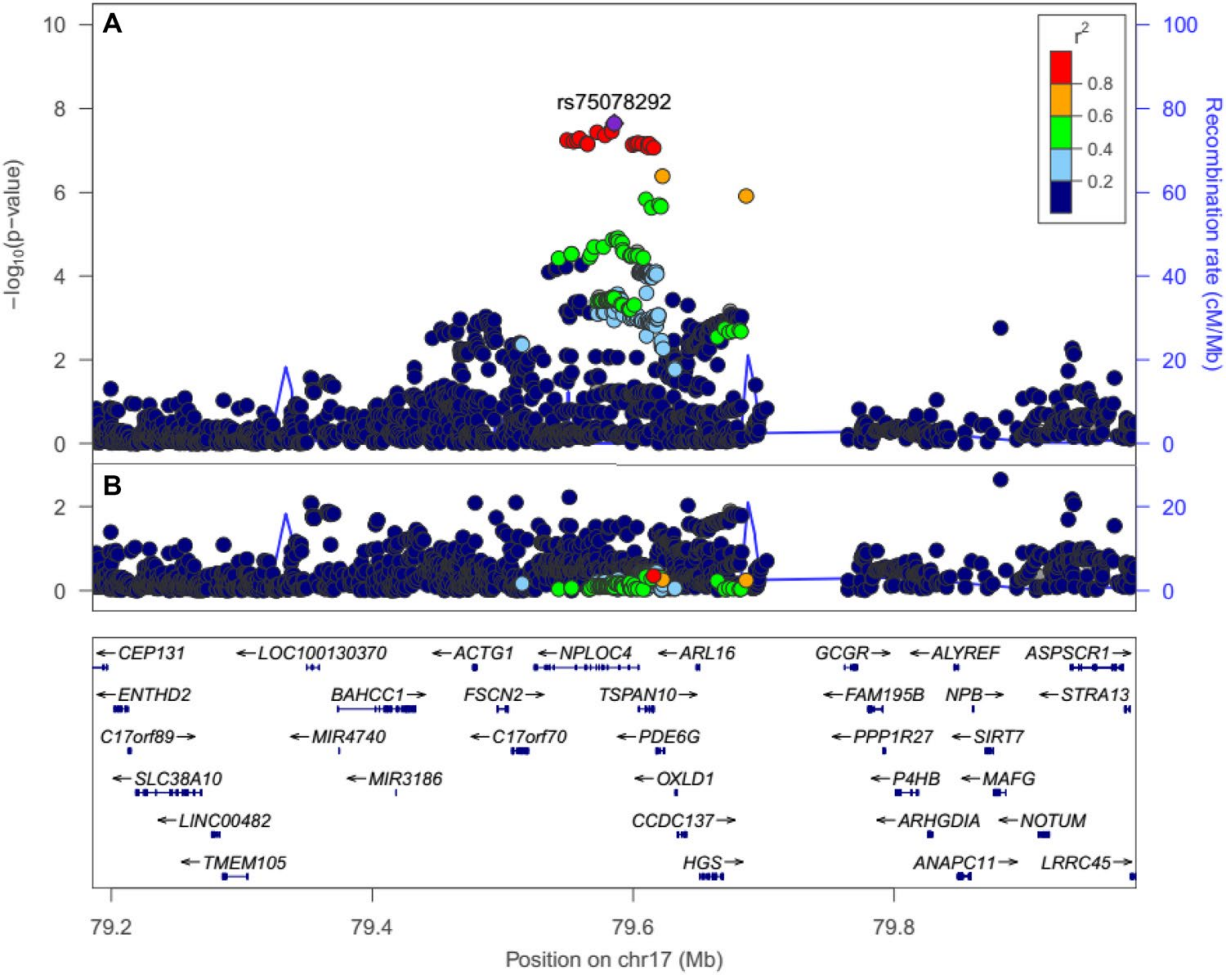
Regional association plot for self-reported strabismus in the region of strongest association, before (**a**) and after (**b**) conditioning on the lead variant

**Fig. 5:**
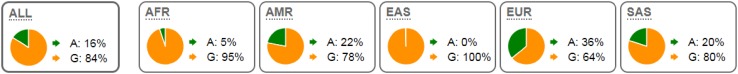
Allele frequency spectrum and of lead GWAS variant across ancestry groups. Allele frequencies for rs75078292. Abbreviations: AFR, Africans; AMR, Native Americans; EAS, East Asians; EUR, Europeans; SAS, South Asians). The results were obtained from *Ensembl* (Yates et al. 2016) for participants of the 1000-genomes project (The 1000 Genomes Project Consortium 2012)

Conditional analysis suggested that a single variant in the region was driving the association at the *NPLOC4–TSPAN10–PDE6G* locus (Fig. 4). To investigate this further, we used the FINEMAP Bayesian fine-mapping approach developed by Benner et al. (2016), which exploits a shotgun stochastic search algorithm to greatly increase computational performance when evaluating the evidence for multiple causal variants in a genomic region. However, this analysis was not able to infer the precise causal variant(s) from amongst the approximately 20 variants in almost perfect LD. Since the associated variants at the locus have widely varying MAF across ancestry groups (Fig. 5), future fine-mapping efforts may be more successful in populations of non-European ancestry.

### Mode of inheritance attributable to the NPLOC4–TSPAN10–PDE6G locus

We explored if an additive, dominant or recessive model best described the relationship between rs75078292 genotype and strabismus. An additive model provided a better fit than a dominant model (*p *= 4.76E−06) while a recessive model provided a better fit than the additive model (*p *= 8.10E−05). The same pattern of results was evident after including the presence/absence of amblyopia, refractive error, astigmatism, and/or anisometropia as covariates in the analysis (Table 2).

**Table 2:** Comparison of modes of inheritance of lead variant at the *NPLOC4–TSPAN10–PDE6G* locus for association with self-reported strabismus in UK Biobank participants (*n* = 66,694)

Covariates included in model	Additive model			Dominant model			Recessive model		
	OR	95% CI	*p* value	OR	95% CI	*p* value	OR	95% CI	*p* value
Baseline	1.24	1.14–1.34	8.92E−08	1.16	1.04–1.29	1.03E−02	1.62	1.41–1.86	1.01E−11
Baseline + amblyopia status	1.16	1.08–1.26	1.70E−04	1.11	0.99–1.24	7.66E−02	1.42	1.23–1.64	2.16E−06
Baseline + refractive error	1.26	1.16–1.36	2.24E−08	1.19	1.06–1.33	3.04E−03	1.64	1.42–1.89	1.61E−11
Baseline + anisometropia	1.23	1.13–1.33	2.97E−07	1.16	1.03–1.29	1.13E−02	1.58	1.37–1.82	1.65E−10
Baseline + astigmatism	1.21	1.12–1.31	2.01E−06	1.13	1.01–1.26	3.45E−02	1.57	1.36–1.81	4.47E−10
Baseline + myopia status	1.25	1.16–1.35	1.71E−08	1.17	1.05–1.31	5.39E−03	1.66	1.44–1.90	1.25E−12
Baseline + anisometropia status	1.22	1.13–1.32	7.28E−07	1.15	1.03–1.29	1.38E−02	1.55	1.35–1.78	8.50E−10
Baseline + astigmatism status	1.22	1.12–1.32	1.07E−06	1.14	1.02–1.28	2.23E−02	1.57	1.36–1.81	3.77E−10

Baseline model included covariates: age, sex, first 10 PC, genotyping array; covariate definitions. Refractive error = mean spherical equivalent refractive error in diopters averaged between the two eyes; anisometropia = anisometropia in diopters coded as a continuous variable; astigmatism = refractive astigmatism in diopters averaged between the two eyes; myopia status = binary variable coded as zero unless refractive error averaged between the two eyes ≤ −0.5 D; anisometropia status = binary variable coded as zero unless anisometropia ≥ 1.00 D; astigmatism status = binary variable coded as zero unless refractive astigmatism averaged between the two eyes ≥ 1.00 D

Under the recessive model, the risk of strabismus in the 14.8% of the sample homozygous for the minor allele of rs75078292 was OR = 1.62 (95% CI 1.41–1.86, *p *= 1.01E−11). This suggested that the population-attributable risk due to the locus was approximately 8.4%.

### Co-localisation of association signals for strabismus and refractive error

The *NPLOC4–TSPAN10–PDE6G* locus was identified in a previous GWAS for self-reported myopia by Pickrell et al. (2016) carried out using data for individuals of European ancestry from the personal genomics company 23andMe. The variant most strongly associated with refractive error in the Pickrell et al. study was rs9747347, which is in perfect LD with the lead strabismus variant (*r*^2^ = 1.00). In analyses adjusted for age, sex, genotyping array and the first 10 PCs, we found that the strabismus risk allele of rs75078292 was also associated with myopia (OR = 1.06, *p *= 5.78E−07) and refractive error (regression coefficient = − 0.09 D, 95% CI − 0.12 to − 0.06, per copy of the risk allele, *p *= 2.77E−08) in UK Biobank participants consistent with the perfect LD between rs9747347 and the similar frequencies of their respective risk alleles. Adjusting for amblyopia or strabismus had minimal impact on the magnitude of the association between rs75078292 and either myopia or refractive error.

After stratifying the sample into myopic, emmetropic and hyperopic subsamples (Table 3), there was no longer evidence of an association between rs75078292 genotype and strabismus in the myopic stratum (OR = 1.11, *p *= 6.19E−01), whereas the association in the hyperopic stratum remained strong (OR = 1.64, *p *= 8.30E−07). Association was evident in emmetropes (OR = 1.88, *p *= 7.24E−03) but the statistical support was weaker than in hyperopes, likely due to the difference in the prevalence of strabismus between these subsamples (0.8% in emmetropes vs. 5.0% in hyperopes). The stratified analysis therefore provided further evidence that the association between rs75078292 and strabismus was not driven by refractive error.

**Table 3:** Association of lead variant for strabismus at the *NPLOC4–TSPAN10–PDE6G* locus with self-reported strabismus after stratifying by refractive status

Stratum	Covariates	Cases/controls	Additive model			Recessive model		
			OR	95% CI	*p* value	OR	95% CI	*p* value
Myopes	Baseline	162/20,473	1.15	0.93–1.44	1.99E−01	1.11	0.73–1.70	6.19E−01
Emmetropes	Baseline	104/17,705	1.22	0.93–1.60	1.51E−01	1.88	1.19–2.99	7.24E−03
Hyperopes	Baseline	807/16,217	1.28	1.15–1.42	3.19E−06	1.64	1.35–1.99	8.30E−07
Myopes	Adjusted^a^	162/20,473	1.15	0.92–1.44	2.09E−01	1.11	0.72–1.70	6.44E−01
Emmetropes	Adjusted^a^	104/17,705	1.18	0.89–1.55	2.51E−01	1.77	1.10–2.84	1.96E−02
Hyperopes	Adjusted^a^	807/16,217	1.18	1.06–1.31	2.26E−03	1.47	1.20–1.80	1.86E−04

Participants were classified into refractive error categories based on their spherical equivalent refractive error in each eye meeting the following criteria: myopes: ≤ − 0.5 D; emmetropes: > − 0.5 D and ≤ + 1.0 D; hyperopes: > + 1.0 D. Note that participants whose two eyes were not classified into the same group (e.g. one eye myopic and one eye emmetropic) were excluded. The baseline covariates age, sex, genotyping array, and the first 10 PCs were included in all models

^a^Adjusted for baseline covariates plus presence/absence of amblyopia, presence/absence of strabismus, and anisometropia (D)

### Functional evaluation of the 17q25.3 locus

Notably, the lead variant at the strabismus locus, rs75078292, is in very tight LD (*r*^2^ = 0.98) with non-synonymous variant rs6420484, which introduces a tyrosine residue in place of an evolutionarily conserved cysteine at position 177 of *TSPAN10*, the gene encoding tetraspanin-10 (Online Resource 4). The C177Y substitution would be expected to have adverse functional consequences, given its scaled CADD score of 16.3 (Table 4). The strabismus lead variant rs75078292 and the C177Y-associated variant rs6420484 are also in extremely high LD with an indel, rs397693108, also predicted to have functional consequences (CADD score 16.1). The rs397693108 indel introduces a 4-bp deletion in the mRNA for 2 isoforms of tetraspanin-10, which would result in a frameshift in the coding region. For a third isoform, the indel is predicted to result in nonsense-mediated decay (NMD) of the mRNA. The risk alleles of the C177Y-associated variant rs6420484 and indel rs397693108 have the same MAF, which given their high LD (*r*^2^ = 0.98) implies they occur on the same haplotype. CADD scores for other variants in LD with the lead GWAS variant were lower (Table 4).

**Table 4:** CADD scores and eQTL effects for *TSPAN10* gene expression in cerebellum for variants surpassing the genome-wide significance threshold and in very high LD (*r*^2^ > 0.95) with the lead variant at the locus

SNP	CHR	BP	REF	ALT	GWAS OR	GWAS *p* value	MAF	Scaled CADD score	eQTL *p* value	eQTL effect size
rs6420484	17	79612397	A	G	1.21	8.24E−10	0.35	16.29	3.30E−21	0.84
rs397693108	17	79614932	TTAAC	T	1.21	6.90E−10	0.35	16.08	3.30E−21	0.84
rs9895741	17	79603831	A	G	1.21	1.43E−09	0.35	10.84	3.30E−21	0.84
rs9747347	17	79606820	T	C	1.21	1.27E−09	0.35	7.09	1.80E−21	0.84
rs7405453	17	79615572	A	G	1.21	5.02E−10	0.35	6.92	3.30E−21	0.84
rs62075722	17	79611271	A	G	1.21	8.48E−10	0.35	6.29	3.30E−21	0.84
rs67050149	17	79557043	C	A	0.83	1.25E−09	0.35	5.98	3.30E−21	− 0.84
rs11656126	17	79564542	G	A	0.83	7.96E−10	0.35	5.98	3.00E−21	− 0.84
rs8081701	17	79599441	T	C	1.21	1.47E−09	0.35	2.41	5.40E−21	0.86
rs11650127	17	79572253	G	A	0.83	6.00E−10	0.35	1.85	1.70E−20	− 0.84
rs71373084	17	79564930	C	G	0.83	8.03E−10	0.35	1.65	3.40E−21	− 0.84
rs9905786	17	79602063	G	T	1.21	1.44E−09	0.35	1.08	7.20E−22	0.86
rs112364254	17	79578287	G	A	0.82	4.93E−10	0.35	1.04	2.70E−20	− 0.82
rs7503894	17	79583473	T	C	1.21	5.94E−10	0.35	0.64	3.30E−21	0.84
rs12953229	17	79554271	G	A	0.83	9.50E−10	0.35	0.48	3.30E−21	− 0.84
rs75078292	17	79585492	A	G	1.21	7.95E−10	0.35	0.38	3.20E−21	0.84
rs62075723	17	79611326	G	A	1.21	5.91E−10	0.35	0.15	3.30E−21	0.84
rs34635363	17	79549250	G	A	0.83	9.34E−10	0.35	0.13	3.30E−21	− 0.84
rs12948708	17	79558741	G	A	0.83	9.34E−10	0.35	0.02	3.30E−21	− 0.84

**Fig. 6:**
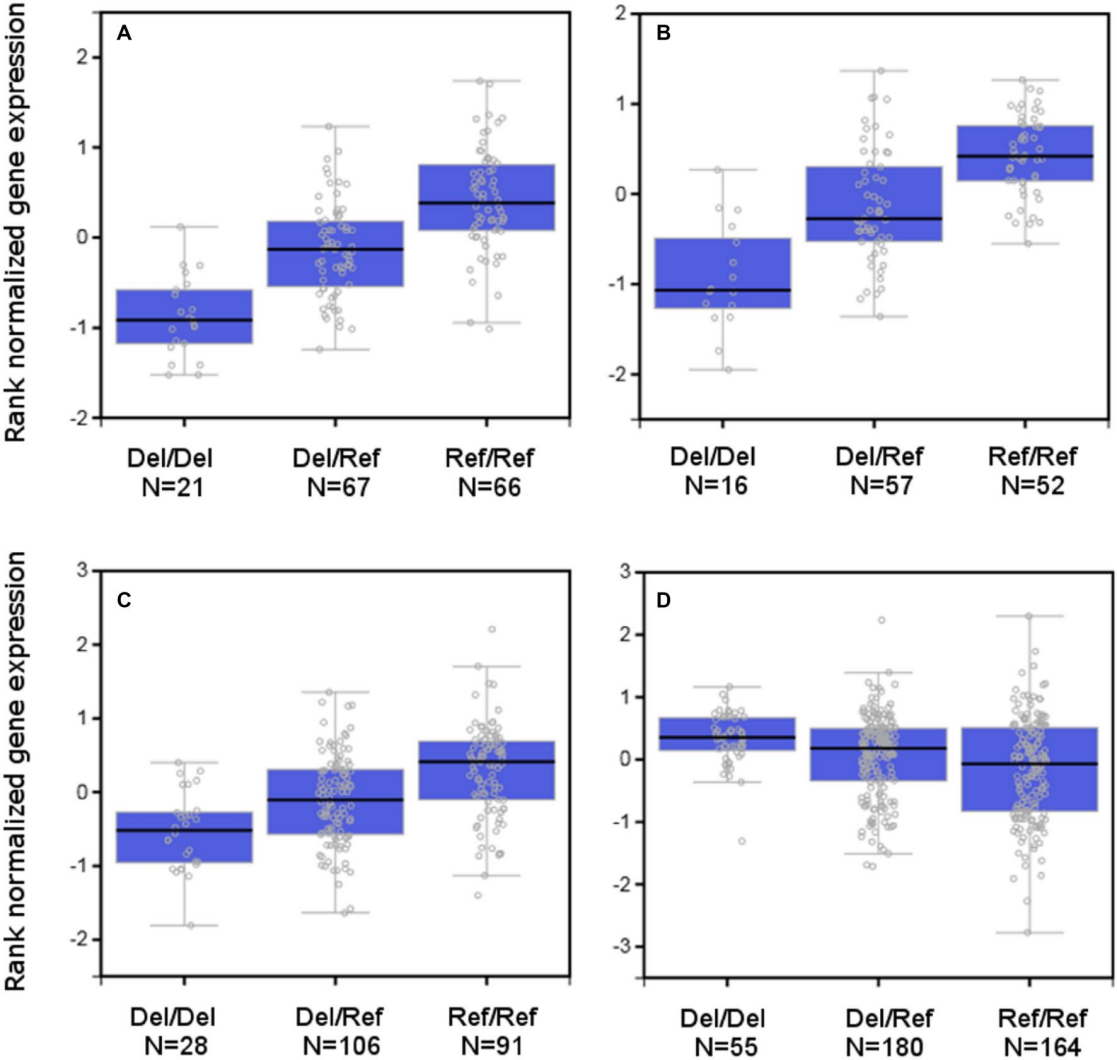
Strabismus-associated indel rs397693108 is an eQTL. Gene expression levels of tetraspanin-10 mRNA in cerebellum (**a**) and human cerebellar hemisphere (**b**), and of phosphodiesterase 6G in testis (**c**), and of ADP ribosylation factor like GTPase 16 in thyroid (**d**) for individuals with the indicated rs397693108 genotype (Ref allele = TTAAC, Del allele = T). Numbers indicate the number of donors for each genotype category. Boxes depict the first and third quartile; whiskers extend to the furthest data point up to a maximum of 1.5 times the height of the box

To search for further evidence of a functional effect of the frameshift/NMD-causing indel rs397693108, we examined *TSPAN10* gene expression in tissue samples analysed by the GTEx consortium (The GTEx Consortium 2015). Although the tissues potentially most relevant to strabismus development, such as extraocular muscle, retina and visual cortex, were not amongst the 53 tissues assessed by the GTEx Consortium, the regulation of gene expression is often conserved across tissue types and therefore can still be highly informative (Pierson et al. 2015). In cerebellum and human cerebellar hemisphere the strabismus-associated risk allele was strongly associated with reduced expression of *TSPAN10* (*p *= 1.30E−10 and *p *= 8.10E−12, respectively; Fig. 6a, b) consistent with degradation of an mRNA isoform via NMD. Furthermore, there was evidence for *cis-*eQTL effects at two other genes. In testis, the risk allele was associated with reduced phosphodiesterase 6G gene (*PDE6G*) expression (*p *= 5.82E−06; Fig. 6c), while in thyroid, it was associated with expression of ADP ribosylation factor like GTPase 16 (*ARL16*) mRNA levels (*p *= 2.10E−06; Fig. 6d). In testis, rs397693108 was the 47th-ranked eSNP associated with *PDE6G* expression; however, it had an almost 1.6-fold lower effect than the top-ranked variant located 15 kb distant (rs11150804, *p* = 1.80–41). Like *PDE6G*, the *ARL16* gene encoding ADP ribosylation factor like GTPase 16 is located nearby to *NPLOC4* and *TSPAN10* (Fig. 4). In thyroid, rs397693108 was the 232nd ranked eSNP associated with *ARL16* expression, and had a more than fourfold lower effect than the top-ranked variant situated 34.5 kb away (rs7503637, *p* = 5.50E−96). By contrast, rs397693108 was the 15th highest-ranked *TSPAN10* eQTL in cerebellum and had an effect size equal to the top-ranked variant (rs9905786, *p* = 7.20E−122). In cerebellar hemisphere tissue rs397693108 was the 63rd-ranked *TSPAN10* eQTL.

Structural variants (SVs) are also known to regulate gene expression, including at the 17q25.3 locus where Chiang et al. (2017) found evidence for co-regulation of *TSPAN10* gene expression by SVs and SNPs. Thus, in summary, there was strong evidence that the causal variant underlying the association with strabismus acts as a *cis*-eQTL for *TSPAN10* in neural tissue, and for *PDE6G* and *ARL16* in certain other tissues. Of these three eGenes, the evidence suggested *TSPAN10* as the most likely causal eGene. However, since gene expression can be highly tissue-specific, further functional studies will be required to examine if eQTL effects are important in the mechanism by which this locus influences strabismus development.

### Independent replication in a clinician-diagnosed sample of children

We sought independent replication of the findings from UK Biobank in a sample of 7-year-old children (*n* = 5200) from the ALSPAC cohort who were clinically assessed for strabismus by an experienced orthoptist. The results are presented in Table 5. The association with strabismus was replicated in the clinician-diagnosed sample of children (Table 5). For any manifest strabismus, the OR = 1.44 (95% CI 1.11–1.88, *p* = 0.007) and OR = 1.85 (95% CI 1.16–2.95, *p* = 0.009) for additive and recessive models, respectively, were consistent with UK Biobank in terms of risk allele, effect size and likely mode of inheritance. Adjusting for amblyopia in these analyses had negligible impact (Table 5). It was notable that the association with strabismus appeared to be restricted to exotropia (divergent strabismus). However, some of the children may have had exotropia resulting from surgery to correct esotropia, hence this latter result should be interpreted with caution (especially in view of the small number of only 28 children with exotropia in the ALSPAC sample).

**Table 5:** Association of amblyopia and strabismus with lead GWAS variant in ALSPAC replication sample

Phenotype	Sample size cases/controls	Inheritance	Baseline model		Adjusted model	
			OR (95% CI)	*p* value	OR (95% CI)	*p* value
History of strabismus	145/5055	Additive	1.36 (1.07–1.73)	0.011	1.34 (1.04–1.73)	0.026
		Recessive	1.72 (1.12–2.63)	0.013	1.58 (0.98–2.55)	0.061
Manifest strabismus	116/5084	Additive	1.44 (1.11–1.88)	0.007	1.43 (1.07–1.92)	0.016
		Recessive	1.85 (1.16–2.95)	0.009	1.72 (1.00–2.95)	0.050
‘Esotropia’	143/5057	Additive	1.08 (0.85–1.38)	0.542	1.02 (0.78–1.32)	0.910
		Recessive	1.20 (0.74–1.95)	0.450	1.00 (0.59–1.72)	0.989
‘Exotropia’	28/5172	Additive	1.76 (1.04–2.99)	0.035	1.73 (1.02–2.93)	0.040
		Recessive	2.47 (1.05–5.83)	0.039	2.37 (1.00–5.63)	0.051
Amblyopia	189/5011	Additive	1.19 (0.97–1.48)	0.100	0.97 (0.77–1.23)	0.789
		Recessive	1.36 (0.91–2.02)	0.135	1.10 (0.69–1.74)	0.690

Baseline model adjusted for sex. Adjusted amblyopia analysis: adjusted for sex and strabismus. Adjusted strabismus analyses: adjusted for sex and amblyopia

Note ‘Esotropia’ includes large (> 10 pd) esophoria as well as manifest esotropia and ‘Exotropia’ similarly includes large (> 15 pd) exophoria as well as manifest exotropia, hence *N* of esotropia + exotropia is greater than for manifest strabismus, which includes only cases manifest on the day of examination

Phenotypes were ascertained in children at age of 7 years

### Tissue distribution of TSPAN-10 in mouse retina

**Fig. 7:**
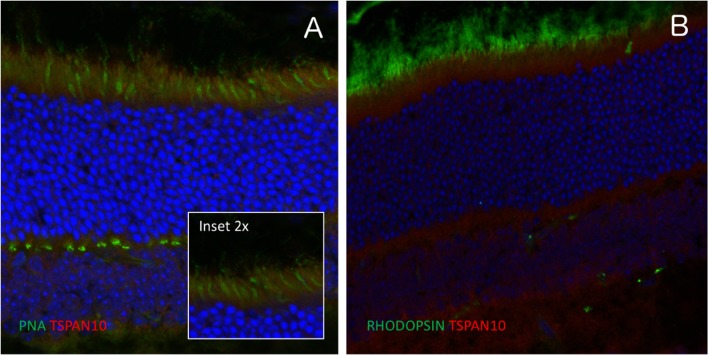
Immuno-localisation of tetraspanin-10 in the mouse retina. **a** Tetraspanin-10 co-localised with Peanut Agglutinin Lectin (PNA), a cone photoreceptor inner and outer segment marker. **b** Tetraspanin-10 did not co-localise with rhodopsin, a rod photoreceptor marker

**Fig. 8:**
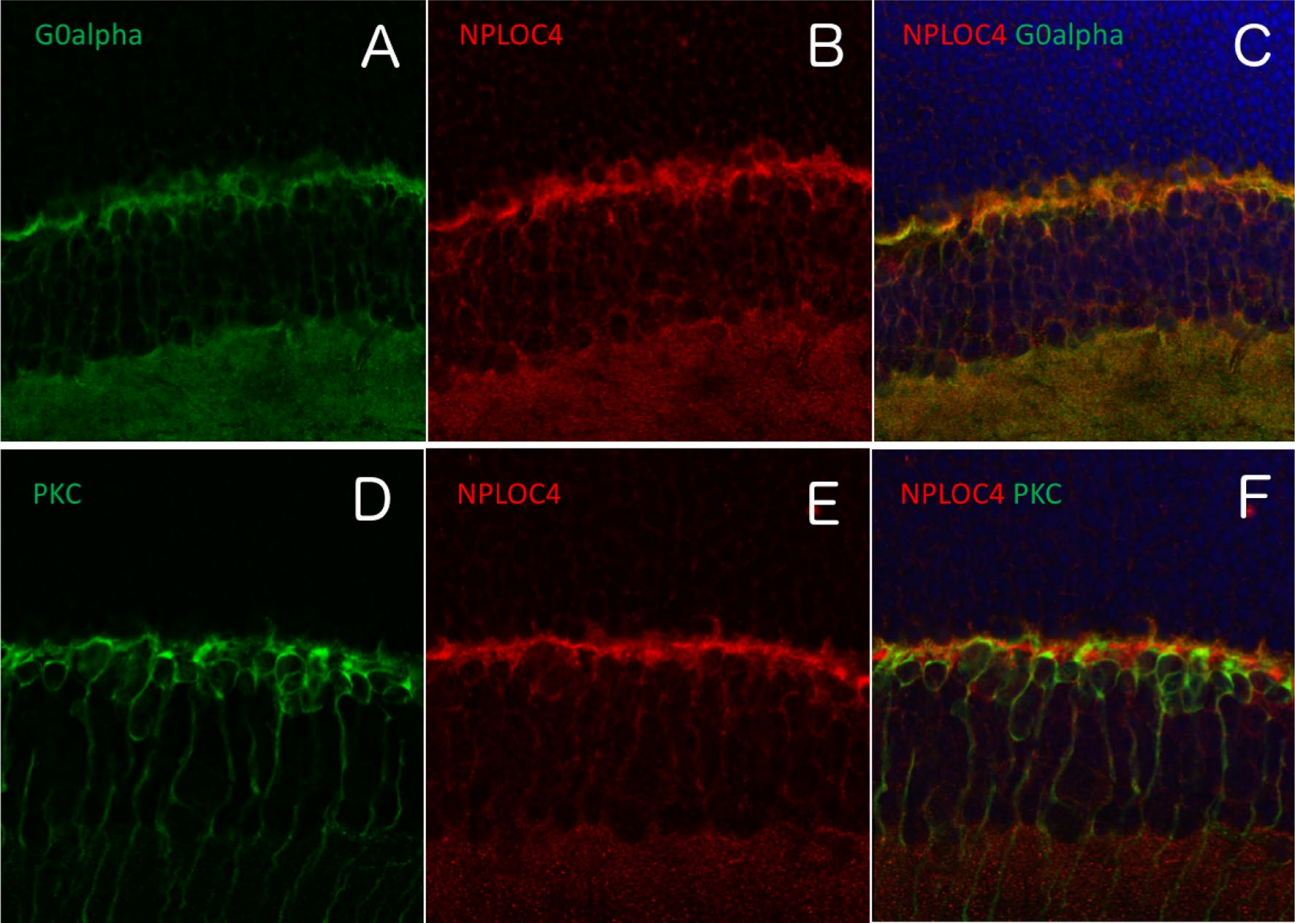
Immuno-localisation of NPL4 homolog, ubiquitin recognition factor (NPLOC4) in the mouse retina. **a**–**c** NPLOC4 co-localised with G0alpha, which a marker for the dendritic tips of ON bipolar interneurons. **d**–**f** NPLOC4 did not co-localise with protein kinase-C (PKC), a marker for rod bipolar interneurons including their dendrites

In the mouse retina, tetraspanin-10 co-localised with Peanut Agglutinin Lectin (PNA), which is a marker of cone photoreceptor inner and outer segments. There was no co-localisation with rhodopsin, a rod photoreceptor marker (Fig. 7). NPL4 homolog, ubiquitin recognition factor (NPLOC4) co-localised with G Protein subunit alpha O1 (G0alpha), which is a marker of ON bipolar cells, but not with Protein kinase-C alpha (PKC), a marker of rod bipolar cells (Fig. 8). This suggested TSPAN10 is expressed in cone photoreceptors and NPLOC4 in ON-cone bipolar cells in the mouse retina. Previous studies have shown that phosphodiesterase 6G (PDE6G) is expressed in both rod and cone photoreceptors (Dvir et al. 2010).

## Discussion

These results provide strong evidence that a commonly occurring polymorphism situated within a high-LD cluster of approximately 20 variants on chromosome 17q25.3 contributes to susceptibility to strabismus. This association with strabismus was independent of the previously documented association with myopia at this locus, since the degree of association with strabismus was minimally attenuated by statistical adjustment for refractive error, myopia status, or anisometropia. These findings suggest a new avenue for strabismus research; namely, to discover how and why gene(s) at the *NPLOC4–TSPAN10–PDE6G* locus influence the visual system’s response to visual experience in childhood. Our results add to the evidence from the GWAS carried out by Shaaban et al. (2018), that commonly occurring variants contribute to susceptibility for strabismus. Notably, the lead variant (rs2244352) associated with non-accommodative esotropia in the Shaaban et al. GWAS was not associated with self-reported strabismus in the current study, suggesting the possibility that the association with rs2244352 is specific to non-accommodative esotropia rather than strabismus more generally.

We reasoned that three criteria should be met in order for a case–control GWAS to detect genetic variants associated with a trait-of-interest. First, the trait must have a genetic component. Second, the sample of ‘controls’ should contain relatively few individuals truly affected by the disorder, i.e. a low proportion of misclassified cases. Third, the sample of ‘cases’ must be enriched for individuals truly affected by the disorder. Regarding our first criterion, recent progress in identifying mutations causing strabismus (Maconachie et al. 2013; Ye et al. 2014; Kruger et al. 2013; Shaaban et al. 2018) provided support for the hypothesis that susceptibility to strabismus has a genetic component. In their pioneering study employing a set of shared controls, the Wellcome Trust Case Control Consortium (2007) carried out simulations demonstrating that statistical power was minimally reduced if fewer than 5% of ‘controls’ were actually misclassified/undiagnosed cases. Given the known prevalence of strabismus in the population (2–4%), selecting controls as participants who did not self-report strabismus was therefore considered an effective approach for meeting our second criterion. To meet our third criterion we sought to confirm that the group of UK Biobank participants who self-reported strabismus was enriched for people with ‘true’ strabismus. To do this, we analysed the level of comorbid traits such as self-reported amblyopia, anisometropia and asymmetric VA in self-reported strabismus cases and controls, which demonstrated convincingly that the comorbidities were indeed over-represented amongst cases (Table 1; Fig. 2). Most importantly, we verified that the association with strabismus was replicated in an independent cohort of 7-year-old children with clinician-diagnosed strabismus (OR = 1.85, 95% CI 1.16–2.95).

There was suggestive evidence that the association with strabismus was restricted to non-myopic individuals, and—counter-intuitively—to individuals with exotropia rather than esotropia. To further dissect these inter-relationships will require the collection and genotyping of large case–control samples, or Biobank-scale cohorts, of individuals with clinician-diagnosed strabismus. In view of the worldwide differences in allele frequency of the lead variant at the *NPLOC4–TSPAN10–PDE6G* locus (Fig. 5), GWAS analyses in other ancestry groups should prove fruitful.

Genetic variants at the *NPLOC4–TSPAN10–PDE6G* locus have been associated with a diverse range of ocular phenotypes. As well as the previously mentioned association of variants at the novel strabismus locus being associated with spherical refractive error, Shah et al. (2018) recently reported that these variants were also associated with corneal and refractive astigmatism (independently of spherical refractive error), while Gao et al. (2019) reported that rs7405453, which is in perfect LD with the lead strabismus variant rs75078292 (*r*^2^ = 1.0) was associated with macular thickness. Two SNPs within *NPLOC4* (rs6565597 and rs9894429) are associated with age-related macular degeneration (AMD) and eye colour, respectively (Fritsche et al. 2015; Liu et al. 2010). Missense variant rs201259422, which is located only 2.5 kb from the *TSPAN10* C177Y-associated variant and introduces a D266N amino acid substitution in *TSPAN10*, is associated with microvascular (central retinal venule) diameter in human retina (Jensen et al. 2016). Given the association of variants at the *NPLOC4–TSPAN10–PDE6G* locus and AMD, we carried out a sensitivity analysis examining the association between strabismus and rs75078292 after excluding UK Biobank participants with self-reported AMD. The results were minimally affected (Online Resource 5).

Under the most parsimonious inheritance model—recessive inheritance—the magnitude of the association with strabismus (OR ≈ 1.4–1.8) is much larger than those typically reported in GWAS studies of complex traits (MacArthur et al. 2017). In view of the wide range of *adverse* effects associated with the *NPLOC4–TSPAN10–PDE6G* locus, it is conceivable that the locus has compensatory *beneficial* effects on other traits.

In silico functional prediction suggested *TSPAN10* as the most likely candidate gene at the locus. The tetraspanin gene family encodes a series of glycosylated, 4-pass transmembrane proteins that feature 1 short and 1 long extracellular loop. Through multiple protein–protein interactions involving other tetraspanins, integrins, signalling receptors, immunoglobulins and proteolytic enzymes, tetraspanins are instrumental in forming membrane micro-domains with specific functions (Seipold and Saftig 2016). Mutations in *PRPH2* (also known as *TSPAN22* or *RDS*) and in *ROM1* (also known as *TSPAN23*), which function together in stabilising the structure of photoreceptor outer segment discs, can cause retinitis pigmentosa (Kajiwara et al. 1994), while mutations in *TSPAN12* can cause familial exudative vitreo-retinopathy type 5 (EVR5) (Poulter et al. 2010; Nikopoulos et al. 2010). Tetraspanin-10, which was first detected in a cDNA library from retinal pigment epithelium/choroid and given the name oculospanin (Wistow et al. 2002), is part of the TSpanC8 subfamily (tetraspanins with 8 cysteine residues in their large extracellular domain). TSpanC8 tetraspanins are implicated in the trafficking of ADAM10 and cleavage of Notch, prior to its further processing by γ-secretase (Dornier et al. 2012). Targets of γ-secretase include (Wakabayashi et al. 2009) APP (amyloid precursor protein) and APLP-2 (amyloid beta precursor-like protein-2); the latter protein has been linked to myopia development through an interaction with time spent reading (Tkatchenko et al. 2015). The precise roles of tetraspanin-10 in neuronal tissues such as cranial nerves, retina and brain are not clear. Notably, the cysteine at position 177 of *TSPAN10* affected by rs6420484 is not one of the 8 cysteines in its large extracellular loop, suggesting that the SNP would not directly impair cleavage activity. The *NPLOC4* gene encodes NPL4 homolog, ubiquitin recognition factor. Little is known of the physiological role of this protein, although its predicted functions include metal ion binding, protein binding, and ubiquitin binding, suggesting a role in ubiquitin-dependent catabolism or endoplasmic reticulum/Golgi organisation. *PDE6G* codes for the (inhibitory) γ-subunit of cGMP-phosphodiesterase. As well as its well-known role in rod phototransduction, this protein regulates MAPK (mitogen-activated protein kinase) signalling via GRK2 (G protein-coupled receptor kinase 2) (Wan et al. 2003). Mutations in *PDE6G* are a rare cause of autosomal recessive retinitis pigmentosa (Dvir et al. 2010; Tsang et al. 1996).

In summary, a genome-wide association study for self-reported strabismus in a large sample of unrelated European individuals identified a single, novel locus associated with this trait.

The association with strabismus was replicated in a sample of 7-year-old children with clinician-diagnosed strabismus (OR = 1.8, *p *= 0.009). The strongest candidate functional variants were a non-synonymous SNP, rs6420484, which introduces a C177Y substitution in the *TSPAN10* gene, and a *TSPAN10*-frameshift-inducing 4-bp indel, rs397693108. These variants were associated with reduced *TSPAN10* gene expression in brain tissues, although such eQTL effects were also observed for the adjacent genes *PDE6G* and *ARL16*, suggesting that the risk of strabismus could be mediated through any one or more of these genes. The identification of a common polymorphism conferring susceptibility to strabismus, opens a new avenue for research to understand the causal mechanisms responsible.

## Electronic supplementary material

Below is the link to the electronic supplementary material.

Supplementary material 1 (DOCX 97 kb)
