# Supplementary material for: A commonly occurring genetic variant within the NPLOC4–TSPAN10–PDE6G gene cluster is associated with the risk of strabismus
Source: Hum Genet. 2019 May 9;138(7):723–37. doi: 10.1007/s00439-019-02022-8 (PMC6611893; doi:10.1007/s00439-019-02022-8)
Supplement: Supplementary file 1 — Supplementary material 1 (DOCX 97 kb) [file 439_2019_2022_MOESM1_ESM.docx]

**A commonly-occurring genetic variant within the *NPLOC4-TSPAN10-PDE6G* gene cluster is associated with the risk of strabismus**

**Online Resources**

| **Index** | **Page** |
| --- | --- |
| Online Resource 1. Statistical models for testing the mode of inheritance | S2 |
| Online Resource 2. Lead variants for regions attaining *P* <1.0e-05 in GWAS for strabismus | S3 |
| Online Resource 3. Quantile-quantile plot of GWAS for both traits | S4 |
| Online Resource 4. Evolutionary conservation of cysteine-177 of the TSPAN10 gene. | S5 |
| Online Resource 5. Sensitivity analysis: exclusion of participants with AMD. | S6 |
|  |  |

**Online Resource 1. Statistical models for testing the mode of inheritance**

In order to compare the fit of additive vs. recessive and additive vs. dominant models, genotype data were recoded using the --export AD flag in PLINK2.0.

Dummy variables were defined as follows, in order to fit nested models:

| **Genotype** | **Dom** | **Add** | **Rec** | **dum_rec** | **dum_dom** | **dum_add** |
| --- | --- | --- | --- | --- | --- | --- |
| AA | 0 | 0 | 0 | 0 | 0 | 0 |
| AB | 1 | 1 | 0 | 0 | 1 | 1 |
| BB | 1 | 2 | 1 | 1 | 1 | 0 |

Analyses were conducted in R. For simplicity, covariates are not included in the example script below.

data <- read.table ("………...raw", header=TRUE)

data$dum_rec <- ifelse(data4$SNP==2, 1, 0)

data$dum_dom <- ifelse(data4$SNP >0, 1, 0)

data$dum_add <- ifelse(data4$SNP==1, 1, 0)

model_rec <- glm(Strabismus ~ dum_rec, family=binomial(),data=data)

model_rec_add <- glm(Strabismus ~ dum_rec + dum_add, family=binomial(),data=data)

model_dom <- glm(Strabismus ~ dum_dom, family=binomial(),data=data)

model_dom_add <- glm(Strabismus ~ dum_dom + dum_add, family=binomial(),data=data)

anova(model_rec, model_rec_add)

anova(model_dom, model_dom_add)

**Online Resource 2. Lead variants for regions attaining P <1.0e-05 in GWAS for Strabismus.** Abbreviations: REF=reference allele; EFFECT=effect allele; MAF=minor allele frequency; INFO=IMPUTE2 imputation quality INFO metric; OR=odds ratio; CI=confidence interval.

| **SNP** | **CHR** | **BP** | **REF** | **EFFECT** | **MAF** | **INFO** | **OR** | **95 %CI** | **P-VALUE** |
| --- | --- | --- | --- | --- | --- | --- | --- | --- | --- |
| rs75078292 | 17 | 79585492 | G | A | 0.35 | 1.00 | 1.25 | 1.16 to 1.36 | 2.24E-08 |
| rs375475939 | 6 | 18594703 | T | C | 0.03 | 1.00 | 1.65 | 1.38 to 1.98 | 7.40E-08 |
| rs116923583 | 16 | 47550308 | A | C | 0.03 | 0.98 | 1.65 | 1.37 to 1.98 | 9.51E-08 |
| rs34349606 | 16 | 34528183 | T | C | 0.03 | 0.99 | 1.66 | 1.37 to 2.02 | 2.90E-07 |
| rs17744237 | 16 | 48245318 | G | A | 0.02 | 1.00 | 1.70 | 1.38 to 2.08 | 3.84E-07 |
| rs117636134 | 10 | 1283600 | G | C | 0.02 | 0.96 | 1.81 | 1.43 to 2.30 | 8.42E-07 |
| rs57229473 | 1 | 246553147 | T | G | 0.07 | 0.99 | 1.42 | 1.24 to 1.63 | 8.77E-07 |
| rs72765677 | 9 | 116461420 | C | T | 0.07 | 1.00 | 0.64 | 0.64 to 0.77 | 1.14E-06 |
| rs79576243 | 5 | 155315050 | T | C | 0.19 | 1.00 | 1.26 | 1.15 to 1.38 | 1.68E-06 |
| rs34203782 | 16 | 35221552 | C | A | 0.03 | 0.98 | 1.60 | 1.32 to 1.94 | 1.96E-06 |
| rs117517710 | 9 | 85060796 | C | T | 0.02 | 1.00 | 1.69 | 1.36 to 2.11 | 2.98E-06 |
| rs2274831 | 10 | 24721946 | A | C | 0.16 | 0.99 | 1.27 | 1.15 to 1.40 | 3.14E-06 |
| rs10932669 | 2 | 217480229 | G | T | 0.13 | 0.98 | 1.29 | 1.16 to 1.44 | 3.34E-06 |
| rs1534560 | 4 | 92172901 | C | T | 0.02 | 1.00 | 1.72 | 1.37 to 2.17 | 4.13E-06 |
| rs116105203 | 5 | 13790138 | T | A | 0.01 | 0.92 | 2.06 | 1.51 to 2.82 | 4.81E-06 |
| rs117682361 | 18 | 33024746 | C | T | 0.02 | 0.94 | 1.62 | 1.32 to 2.00 | 5.10E-06 |
| rs150685865 | 14 | 21754186 | T | C | 0.01 | 0.95 | 1.82 | 1.40 to 2.36 | 5.79E-06 |
| rs186974323 | 6 | 891483 | T | G | 0.01 | 0.97 | 1.94 | 1.45 to 2.58 | 6.34E-06 |
| rs76575122 | 16 | 46920923 | A | C | 0.04 | 0.94 | 1.51 | 1.26 to 1.81 | 6.64E-06 |
| rs143178747 | 9 | 8721155 | G | A | 0.02 | 0.94 | 1.73 | 1.36 to 2.19 | 7.75E-06 |
| rs141951718 | 8 | 3757558 | G | A | 0.01 | 0.94 | 1.92 | 1.44 to 2.57 | 8.31E-06 |
| rs75645041 | 3 | 62553028 | C | G | 0.15 | 0.99 | 1.26 | 1.14 to 1.40 | 9.19E-06 |
| rs142426391 | 13 | 97246273 | C | T | 0.02 | 0.92 | 1.70 | 1.34 to 2.14 | 9.56E-06 |
| rs58174358 | 11 | 120647015 | C | T | 0.04 | 0.98 | 1.54 | 1.27 to 1.27 | 9.76E-06 |

**Online Resource 3. Quantile-quantile plot of GWAS for strabismus.** The x and y axes show expected and observed minus log10 *P*-values for association with the trait.


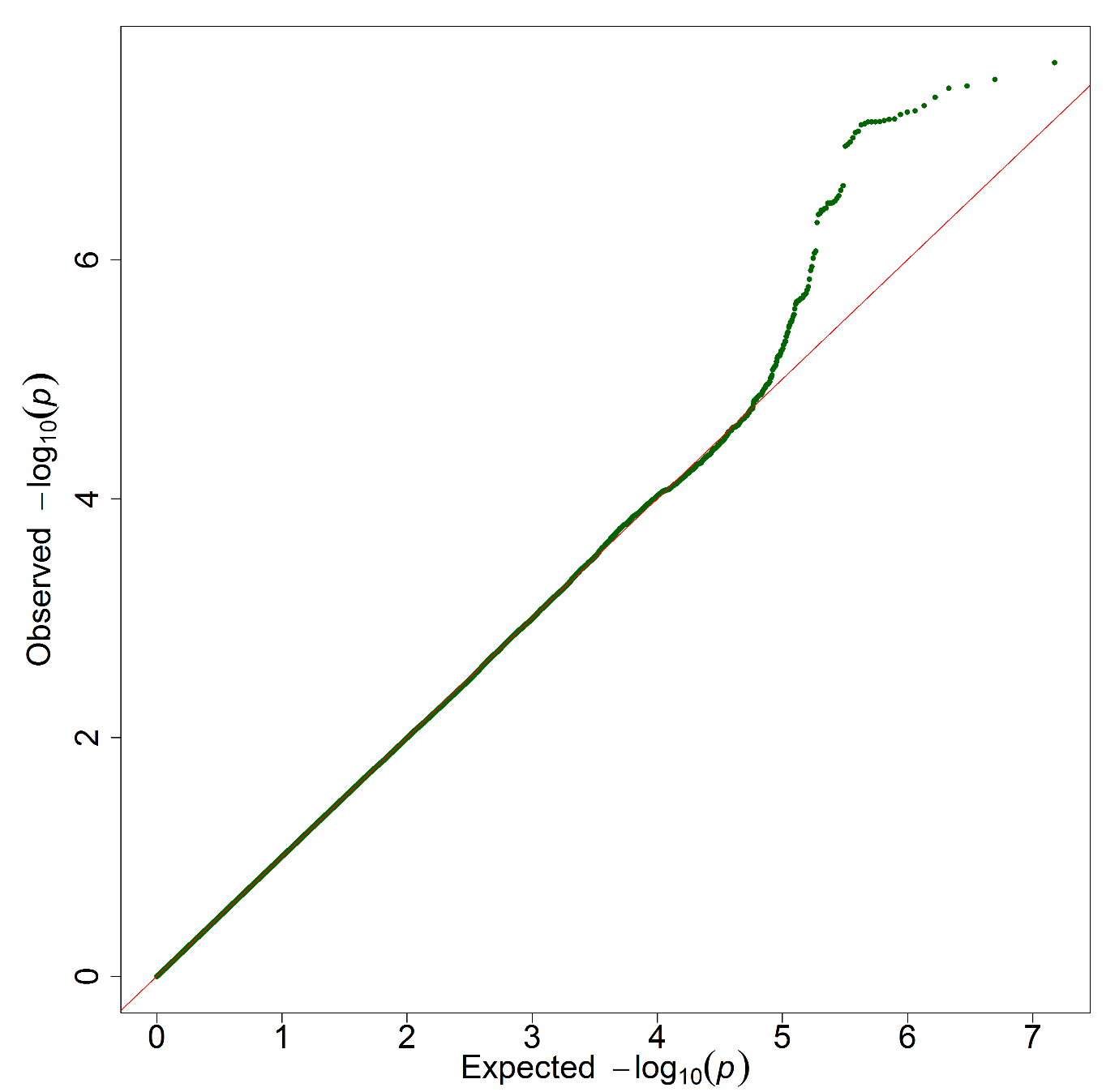


**Online Resource 4. Evolutionary conservation of cysteine-177 of the *TSPAN10* gene.** Protein sequences in the NCBI HomoloGene database were aligned using ClustalW.

| Human | GGLVVSAVSLAG**C**LGALCENTCLLR |
| --- | --- |
| Chimpanzee | GGLVVSAVSLAG**C**LGALCENTCLLR |
| Macaque | GGLVVSTVSLAG**C**LGALCENTCLLH |
| Canine | GGLAVGAVSLAG**C**LGALCENACLLH |
| Bovine | GGLAVSVVSLAG**C**LGALCENTFLLR |
| Mouse | GGLVVSVVSLSG**C**LGAFCENSCLLH |
| Rat | GGLVVSVVSLSG**C**LGAFCENSCLLH |
| Chicken | AGLGASTVSLAG**C**LGVLRSSTCLLR |
| Xenopus | LGLVLALLSMTG**C**IGALRENMCLLR |
| Zebrafish | VGLIVSFLSVSG**C**LGFIRENIYLLR |

**Online Resource 5. Sensitivity analysis: exclusion of participants with AMD.** The association between strabismus and rs75078292 genotype was repeated after excluding participants who self-reported having age-related macular degeneration. The sample size was n=65,917 participants (1,325 cases and 64,592 controls). Age, sex, genotyping array and 10 PCs were included as covariates.

| **Inheritance model** | **OR** | **95%CI** | ***P*-value** |
| --- | --- | --- | --- |
| Additive | 1.24 | 1.15 1.34 | 8.16E-08 |
| Recessive | 1.63 | 1.42 1.87 | 8.45E-12 |
| Dominant | 1.16 | 1.04 1.30 | 1.03E-02 |
